# Surgical Outcomes of Epiretinal Human Amniotic Membrane Transplantation for Refractory Macular Holes

**DOI:** 10.3390/jcm15041443

**Published:** 2026-02-12

**Authors:** Sibel Doguizi, Cemile Ucgul Atilgan, Kemal Tekin

**Affiliations:** Department of Ophthalmology, Ulucanlar Eye Training and Research Hospital, Ankara 06230, Türkiye; cemileucgul@ymail.com (C.U.A.); kemal_htepe@hotmail.com (K.T.)

**Keywords:** epiretinal transplantation, human amniotic membrane, pars plana vitrectomy, refractory macular hole

## Abstract

**Background/Objectives**: Refractory macular holes (MHs) that persist after conventional internal limiting membrane (ILM) peeling pose a significant surgical challenge. In this study, we analyzed the anatomical and functional outcomes of epiretinal human amniotic membrane (hAM) transplantation in patients with MHs. **Methods**: This retrospective study included 10 eyes of 10 patients with refractory MHs. All patients underwent 25-gauge pars plana vitrectomy, epiretinal cryopreserved hAM transplantation, and C3F8 gas tamponade. The large hAM graft was placed over the macula with the stromal side facing the retina. Preoperative and postoperative best-corrected visual acuity (BCVA), optical coherence tomography (OCT) findings, and MH dimensions were recorded. **Results**: The mean follow-up period was 7 months (range: 3–14 months). The mean preoperative minimum linear diameter and base diameter of the MHs were 715 ± 212 μm and 1114 ± 258 μm, respectively. Anatomical closure was achieved in all patients (100%). Postoperative OCT revealed rearrangement of the inner and other retinal layers in 7 out of 10 patients (70%), with partial restoration of the outer retinal layers. The mean logMAR BCVA improved significantly from 1.60 ± 0.37 preoperatively to 1.00 ± 0.45 postoperatively (*p* < 0.001). No graft dislocation, rejection, or other significant complications were observed. **Conclusions**: Our preliminary results suggest that epiretinal human amniotic membrane transplantation is a feasible and promising surgical technique for achieving anatomical closure and functional improvement in refractory macular holes in which conventional ILM peeling has failed.

## 1. Introduction

Idiopathic full-thickness macular holes (MHs) are significant vitreoretinal pathologies affecting all layers of the neurosensory retina in the foveal region, leading to severe reduction in central visual acuity, metamorphopsia, and central scotoma [1]. Until recent years, the combination of pars plana vitrectomy (PPV), internal limiting membrane (ILM) peeling, and gas tamponade was considered the “gold standard” for the surgical management of these cases [2]. Although this approach provides anatomical closure rates of 90% to 95%, particularly in holes smaller than 400 μm and stage 2–3 holes, success rates may decrease in cases with large diameters (>400 µm), chronic duration, or traumatic etiology [3,4]. More importantly, “refractory” macular holes that fail to close or recur despite primary surgery and ILM peeling remain one of the most complex challenges in vitreoretinal surgery [5,6].

In the management of refractory cases, the absence of residual ILM tissue around the hole (the “No-ILM” situation) limits surgical options [7]. Various reconstructive techniques, such as autologous neurosensory retinal transplantation (ART), lens capsule flaps, extended ILM peeling, or relaxing retinotomy, have been proposed to achieve anatomical closure in cases where standard techniques are insufficient [8,9,10]. However, some of these methods may pose disadvantages such as surgical difficulty, potential risk of retinal damage, visual field loss, or limited functional gain [3]. Furthermore, aggressive ILM peeling itself can disrupt retinal morphology by causing iatrogenic trauma, such as the appearance of a “dissociated optic nerve fiber layer” (DONFL) on the inner retinal surface [2,11]. Therefore, particularly in persistent cases, there is a need for “scaffold” materials that not only provide mechanical closure but also biologically support tissue regeneration [12].

Human amniotic membrane (hAM) stands out as a potent biomaterial in regenerative medicine due to its thick basal membrane, avascular stroma, and richness in growth factors (TGF-β, bFGF, EGF, and NGF) [13,14]. Long used in ophthalmology for ocular surface reconstruction due to its low immunogenicity, anti-inflammatory properties, and fibrosis-suppressing structure, hAM has recently become a promising “salvage” treatment option in vitreoretinal surgery [2,15]. It has been reported that hAM provides excellent growth support for retinal pigment epithelium (RPE) cells, facilitates glial cell migration, and promotes the restoration of outer retinal layers by secreting neurotrophic factors [16,17].

In the literature, hAM is applied either as a plug (inlay) placed inside the hole or as a patch (overlay) placed over the retina [5]. Although the plug technique has been found to be successful in some studies, the difficulty of fitting the graft size exactly to the hole and the potential risk of RPE damage are debated [18]. In contrast, the epiretinal patch (overlay) technique is preferred for its minimal interference with the natural anatomical structure of the retina, preserving RPE integrity, and providing biological support over a larger surface area during the healing process [19,20]. Recent studies demonstrate that hAM transplantation yields high anatomical closure rates (93–100%) not only in idiopathic refractory holes but also in much more challenging scenarios such as high myopia-associated macular holes, cases complicated by retinal detachment, and traumatic holes [21,22,23]. Additionally, systematic reviews and meta-analyses have reported that hAM use presents low complication rates and offers significant visual function improvement in refractory cases [2,24].

The aim of this study is to evaluate the anatomical closure success, the healing process of outer retinal layers (ellipsoid zone and external limiting membrane), and the functional outcomes of epiretinal human amniotic membrane transplantation in refractory macular holes that failed to close despite conventional ILM peeling. Through our study, we specifically aim to document the feasibility and potential regenerative effects of hAM in an environment devoid of an ILM.

## 2. Methods

### 2.1. Study Design and Ethics

This retrospective study was conducted at the Department of Ophthalmology, Ulucanlar Eye Training and Research Hospital. The study protocol adhered to the tenets of the Declaration of Helsinki and was approved by the Numune Training and Research Hospital (E-37-1685). Written informed consent was obtained from all participants prior to the surgery, following a detailed explanation of the procedure and potential risks.

### 2.2. Patient Selection

We reviewed records of patients who underwent surgery for refractory MHs (March 2023–April 2025). The inclusion criteria were the presence of a persistent full-thickness MH despite previous PPV with ILM peeling and intraocular gas tamponade. The exclusion criteria included a history of retinal vascular occlusion, diabetic retinopathy, retinal neovascularization, or severe ocular trauma. A total of 10 eyes of 10 patients met the inclusion criteria.

### 2.3. Ophthalmic Examination and OCT Analysis

All patients underwent a comprehensive ophthalmic examination, including measurement of best-corrected visual acuity (BCVA) and slit-lamp biomicroscopy. BCVA was measured using a Snellen chart and converted to the logarithm of the minimum angle of resolution (logMAR) for statistical analysis.

Retinal images were acquired using spectral-domain optical coherence tomography (SD-OCT) (Spectralis, Heidelberg Engineering GmbH, Heidelberg, Germany). The scanning protocol included posterior pole images centered on the fovea (61 acquisitions with 120 μm intervals) and high-resolution six-line radial scans. All measurements were performed using the built-in caliper function of the OCT device on the cross-sectional image passing through the foveal center.

The Minimum Linear Diameter (MLD) was defined as the narrowest horizontal width of the macular hole at the mid-retinal level, and the Base Diameter (BD) was defined as the width of the hole at the level of the RPE. Anatomical closure was defined as the complete reapproximation of the neurosensory retina over the fovea (Type 1 closure).

To ensure objectivity, all preoperative and postoperative OCT parameters (including MLD, BD, and ELM/EZ defects) were measured by two independent experienced graders (S.D. and K.T.) who were masked to the patient’s identity and clinical outcomes. The average of the two measurements was used for analysis.

### 2.4. Preparation of Human Amniotic Membrane

Human amniotic membrane grafts were prepared under sterile conditions from placentae obtained shortly after elective cesarean delivery. The collection of placentae was approved by the local Ethics Committee. Donor serological screening was performed to rule out transmissible infections, including human immunodeficiency virus (HIV), hepatitis B and C viruses, and syphilis. The placenta was initially washed with sterile saline solution several times to remove blood clots. The amniotic membrane was then carefully separated from the chorion to isolate the pure amniotic membrane layer and rinsed with sterile saline solution supplemented with 100 U/mL benzyl penicillin, 200 μg/mL ciprofloxacin, and 2.5 μg/mL amphotericin B [13,14]. Following incubation in this antibiotic-containing saline solution for 24 h, the amniotic membrane was flattened on a sterile ophthalmic drape, cut into sections (typically 2 × 3 cm), and stored in plates containing a 1:1 mixture of glycerol and Dulbecco’s Modified Eagle Medium at −80 °C (cryopreservation) until further use. On the day of surgery, the graft was retrieved from storage and transported to the operating room. The thawed amniotic membrane was placed in a sterile dish and rinsed repeatedly with sterile balanced salt solution to remove the storage medium before intraocular use.

### 2.5. Surgical Technique

All surgeries were performed by the same experienced surgeon using a standard 25-gauge 4-port transconjunctival micro-incision vitrectomy system (Constellation Vision System, Alcon Surgical, Fort Worth, TX, USA). After core vitrectomy, Brilliant Blue G dye was used to stain the retinal surface to confirm the absence of any residual ILM or epiretinal membranes.

A graft diameter of 4 to 8 mm was selected to ensure sufficient overlap with the surrounding retina, aiming to enhance adhesion and stability. The graft was inserted into the vitreous cavity through a 25-gauge trocar. Using end-gripping forceps, the hAM was positioned over the macular hole. Crucially, the graft was oriented with the sticky stromal side facing the retina and the epithelial side facing the vitreous. The orientation was verified intraoperatively by identifying the sticky surface with forceps. Perfluorocarbon liquid (PFCL) was injected over the posterior pole to flatten the graft and ensure stable positioning over the macula. Following a fluid–air exchange, the PFCL was carefully removed, ensuring the graft remained in place. Lastly, a non-expansile concentration of 14% perfluoropropane (C3F8) gas was exchanged for air. Patients were instructed to maintain a face-down position for 3 days postoperatively. All patients received the routine postoperative topical antibiotic and steroid therapy typically administered after vitreoretinal surgery. During follow-up, patients were thoroughly evaluated for intraocular inflammation, and no cases of intraocular inflammatory events were observed.

### 2.6. Outcome Measures and Statistical Analysis

The primary outcome was anatomical closure of the MH confirmed by means of OCT. Secondary outcomes included improvement in BCVA and restoration of retinal layers. Statistical analysis was performed using SPSS, version 25 (IBM Corp., Armonk, NY, USA) software. The normality of data distribution was assessed using the Shapiro–Wilk test. Continuous variables were expressed as mean ± standard deviation (SD). Given the small sample size (n = 10), it is acknowledged that normality testing has limited statistical power. Although the Shapiro–Wilk test did not indicate a significant deviation from normality, the paired Student’s *t*-test was used as the primary statistical method. Additionally, a non-parametric Wilcoxon signed-rank test was performed as a sensitivity analysis to confirm the robustness of the BCVA outcomes. A *p*-value of less than 0.05 was considered statistically significant.

## 3. Results

### 3.1. Demographics and Baseline Characteristics

The study included 10 patients (7 females and 3 males) with a mean age of 56.7 ± 6.1 years (range: 50–68 years). Nine patients presented with idiopathic refractory MHs, with one patient having a persistent MH following surgery for retinal detachment (RD) with macular hole. The mean axial length was 23.24 ± 0.71 mm. The baseline characteristics of the study population are summarized in Table 1.

### 3.2. Anatomical Outcomes

The mean preoperative minimum linear diameter of the MHs was 715 ± 162 μm, and the mean base diameter was 1114 ± 258 μm. Anatomical closure was achieved in all 10 eyes (100%) after a single surgery with hAM transplantation. Regarding safety, no intraoperative or postoperative complications were observed. Specifically, there were no instances of endophthalmitis, retinal detachment, graft rejection, contraction, subretinal migration, or rejection. Furthermore, no cases of elevated intraocular pressure, significant inflammation, cystoid macular edema, or epiretinal membrane recurrence were noted. No patient required reoperation, and no significant cataract progression was observed during the follow-up.

**Figure 1:**
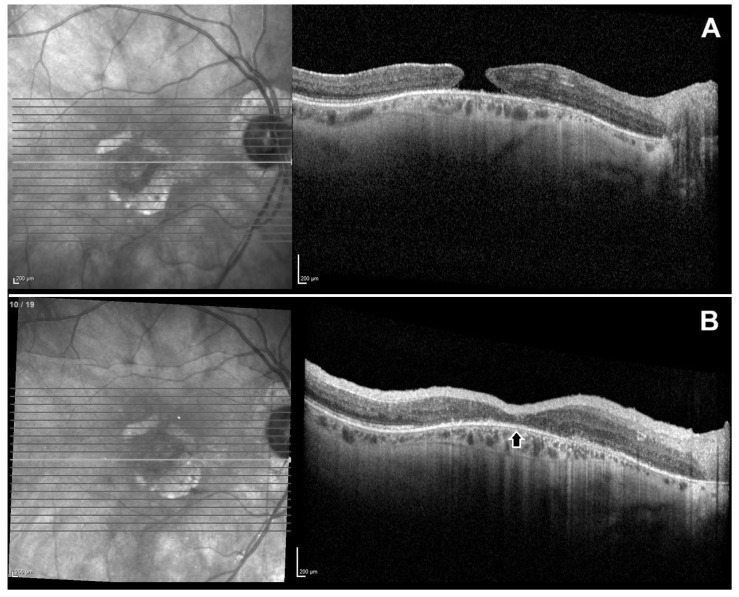
Preoperative and postoperative spectral-domain optical coherence tomography (SD-OCT) images of a patient with refractory macular hole. (**A**) Preoperative OCT scan showing a full-thickness macular hole with atrophic edges. (**B**) Postoperative OCT scan at 2 months showing complete anatomical closure of the hole following human amniotic membrane transplantation. The graft is well-integrated, and there is visible restoration of the foveal contour (arrow).

**Figure 2:**
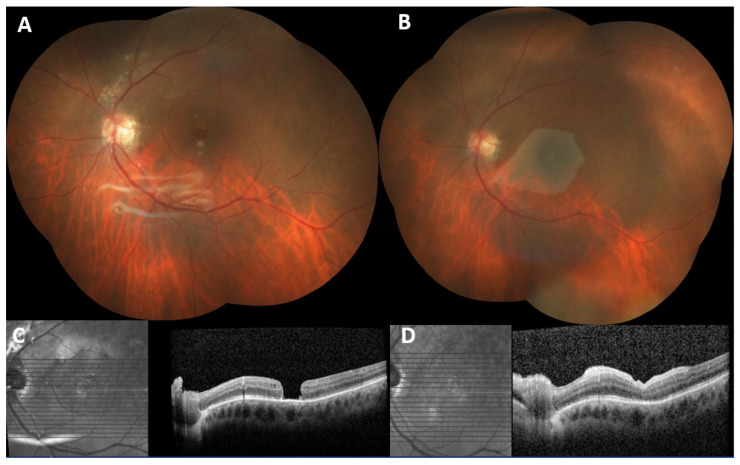
Preoperative and postoperative fundus photographs and spectral-domain optical coherence tomography (SD-OCT) images of a patient with refractory macular hole (after retinal detachment and macular hole surgery with silicon oil). (**A**) Preoperative color fundus photography of the patient; retina attached with intravitreal silicon oil but with refractory macular hole. (**B**) Postoperative fundus photography; large human amniotic patch over the macula. (**C**) Preoperative OCT scan showing a large full-thickness macular hole with atrophic edges. (**D**) Postoperative OCT scan at 2 months showing complete anatomical closure of the hole following epiretinal human amniotic membrane transplantation. The amniotic graft is well-integrated; inner and outer retinal layers are rearranged.

**Figure 3:**
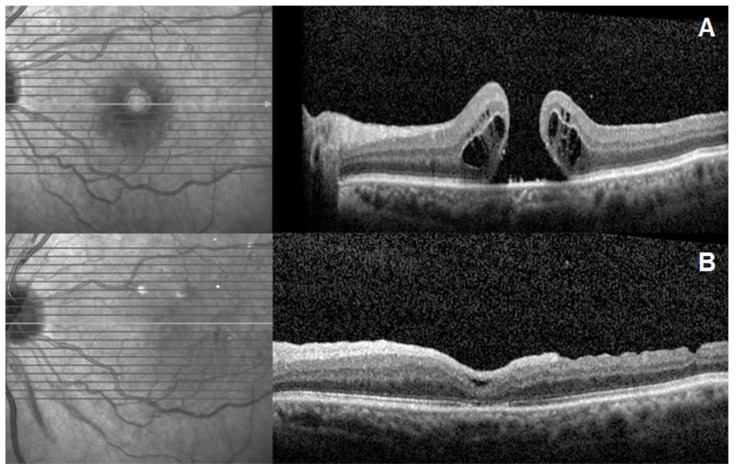
Preoperative and postoperative spectral-domain optical coherence tomography (SD-OCT) images of a patient with refractory macular hole. (**A**) Preoperative OCT scan showing a full-thickness large macular hole with cystoid changes. (**B**) Postoperative OCT scan at 3 months showing complete anatomical closure of the hole; there is visible restoration of the foveal contour and retinal layers.

Postoperative OCT analysis demonstrated the integration of the hAM graft into the retinal defect. Representative cases demonstrating preoperative and postoperative OCT findings are shown in Figure 1, Figure 2 and Figure 3.

### 3.3. Outer Retinal and RPE Integrity Assessment

At baseline, all eyes demonstrated disruption of the ellipsoid zone (EZ) and external limiting membrane (ELM) defects on SD-OCT. At the final follow-up, partial restoration of the ELM and EZ was observed in seven eyes (70%). Quantitatively, the mean EZ defect size decreased from 1216 ± 134 µm preoperatively to 736 ± 311 µm postoperatively and the mean ELM defect size decreased from 1144 ± 123 µm preoperatively to 698 ± 0.101 µm postoperatively. Regarding RPE integrity, no progressive RPE atrophy, hypertransmission defects, or newly developed areas of RPE loss were observed on serial OCT examinations during the follow-up period. Fundus autofluorescence imaging was not routinely available in this retrospective cohort.

### 3.4. Functional Outcomes

The mean follow-up time was 7 months (range: 3–14 months). Visual acuity improved in all patients. The mean preoperative BCVA was 1.60 ± 0.37 logMAR, which significantly improved to 1.00 ± 0.45 logMAR at the final follow-up visit. The improvement in BCVA was statistically significant in both the paired Student’s *t*-test (*p* < 0.001) and the Wilcoxon signed-rank test (*p* = 0.002), confirming the robustness of the observed functional gain. Patients with better baseline visual acuity tended to achieve better final visual outcomes (*p* = 0.012).

When patients were stratified according to MH size, eyes with a minimum linear diameter <700 µm (n = 3) demonstrated a greater mean BCVA gain (−0.53 ± 0.12 logMAR) compared with those with a diameter ≥700 µm (n = 7), which showed a mean improvement of −0.37 ± 0.15 logMAR. Similarly, patients with a shorter MH duration (≤4 months, n = 6) achieved better functional outcomes (mean BCVA gain −0.48 ± 0.14 logMAR) compared to those with longer-standing holes (>4 months, n = 4; −0.32 ± 0.11 logMAR). Although formal statistical comparison was not performed due to the limited sample size, these findings suggest a trend toward greater visual improvement in smaller and shorter-duration refractory macular holes.

## 4. Discussion

Refractory macular holes that fail to close despite standard pars plana vitrectomy and ILM peeling constitute one of the most complex scenarios in vitreoretinal surgery. In these cases, the absence of residual ILM tissue around the hole (the “No-ILM” situation) restricts surgical options. In our study, the 100% anatomical closure rate and statistically significant increase in visual acuity achieved with epiretinal cryopreserved hAM transplantation in this challenging patient group indicate that hAM acts not merely as a mechanical plug but rather as a regenerative scaffold for retinal tissue, contributing to the restoration of foveal architecture. This high anatomical success strongly parallels similar challenging case series in the literature. For instance, Khaqan et al. reported 100% closure using hAM in giant and persistent macular holes [4]; similarly, Calis Karanfil et al. and Ferreira et al. reported full success with a single surgery in refractory cases [13,25]. The results of the multicenter ReMaHo study conducted by Lorenzi et al. also support our findings, confirming that hAM use provides statistically superior closure rates compared to autologous ILM flap techniques, particularly in holes larger than 680 μm [7].

In addition to anatomical success, the biological form and preparation method of the amniotic membrane play a decisive role in surgical outcomes. The efficacy of lyophilized (dried) versus cryopreserved (frozen) grafts is frequently compared in the literature. While Garcin et al. reported an 80% closure rate in their series using a lyophilized amniotic membrane [19], a 100% success rate was achieved in our study with a cryopreserved membrane, aligning perfectly with the results of a comprehensive meta-analysis by Zhang et al.; the authors emphasized that cryopreserved grafts provide a distinct advantage with a 99% closure rate compared to lyophilized grafts (73%) and have a much lower risk of dislocation (3% vs. 26%) [2]. Sharma et al. and Carlà also state that the cryopreservation process better preserves the membrane’s growth factors, anti-inflammatory cytokines, and structural integrity compared to the lyophilized form, thereby strengthening the graft–host tissue interaction [15,26]. Although Huang et al. noted that both graft types could be effective [27], our experience and current literature data suggest that the thicker stromal structure of cryopreserved grafts facilitates surgical manipulation and shortens the learning curve when applied with the “Triple S” (Staining, Sizing, Sliding) technique [28].

Regarding surgical technique, the “epiretinal overlay” (patch) technique preferred in our study is based on the principle of laying the graft over the retina rather than inserting it into the hole, and this approach has been a critical factor in our anatomical and functional outcomes. Teoh and Garcin et al. state that this “overlay” position more effectively respects the natural organization of retinal layers and carries a lower risk of triggering foveal gliosis compared to subretinal plugging [5,19]. Ventre et al. reported that in the plug technique, if the graft is cut larger than the hole diameter, the resulting “centrifugal thrust” can create a mechanical barrier and delay closure [18]. The rapid healing process and orderly organization of retinal layers under the graft in our series confirm the mechanical advantages of epiretinal placement. Furthermore, as suggested by Iannetta et al., placing the graft with the stromal (sticky) side facing the retina increased adhesion [20], preventing graft dislocation and contributing to the absence of graft displacement in any patient in our study. This technique maximizes surgical safety when combined with adjunctive methods such as viscoelastic, PFCL, or intraoperative OCT use, as proposed by Qian and Saab [29]. In the literature, it has generally been reported that using grafts up to 4–6 mm in size is safe, and some reports even suggest that anatomical outcomes might be improved with grafts larger than 6 mm. Additionally, it has been reported that the light-transmitting properties of the amnion seem compatible with visual acuity preservation. Therefore, although we did not adhere to a strict standardized size, we utilized grafts of approximately 4–8 mm placed epiretinally to ensure adequate coverage [26,30,31].

Although anatomical closure in refractory cases does not always translate to full functional recovery, a statistically significant improvement in visual acuity (from logMAR 1.60 to 1.00) was recorded in our study. This finding is consistent with results reported by Proença et al. and Caporossi et al., showing that visual gain is maintained in long-term follow-ups [16,17]. The basis of visual improvement lies in hAM’s neurotrophic factors (NGF, bFGF, EGF) supporting photoreceptor viability and promoting the restoration of outer retinal layers (ellipsoid zone and external limiting membrane) [14]. The outer retinal restoration observed in 70% of cases in our study suggests that hAM is not just a barrier but a biological healer. A comprehensive meta-analysis by Zhang et al. further corroborates our results, reporting significant visual improvement in the majority of refractory cases treated with hAM, thereby validating its functional efficacy [2]. Although Özdemir Zeydanlı et al. reported limited regeneration despite anatomical success in very complex and high myopic cases [22], it appears that functional potential is preserved in isolated refractory holes like those in our series, and hAM unlocks this potential.

Alternative surgical techniques exist for the management of refractory holes; however, hAM transplantation stands out with its safety profile and ease of application. For example, although autologous neurosensory retinal transplantation (ART) offers high anatomical success (94%) as reported by Hanai et al. and Grewal et al. [3,9], it harbors disadvantages such as its invasive nature, potential risk of retinal detachment, peripheral visual field loss, and failure of the graft to fully integrate with surrounding tissue [6,8]. Additionally, it is known that extended ILM peeling maneuvers in refractory cases lead to iatrogenic trauma such as the “dissociated optic nerve fiber layer” (DONFL) appearance on the retinal surface and can leave permanent damage in the inner retinal layers [2,11]. In this context, hAM transplantation is an advantageous “salvage” treatment option as it is less traumatic compared to ART, does not require autologous tissue harvesting, and initiates biological repair without causing additional damage to the retina. Szurman et al. also emphasized that hAM use in revision surgeries is one of the most reliable adjuvant techniques with 100% success [12].

Regarding the safety profile, the absence of complications such as graft rejection, endophthalmitis, chronic hypotony, or severe inflammation in our study can be explained by hAM’s low immunogenicity and potent anti-inflammatory properties [24]. As emphasized in recent comprehensive reviews and expert guidelines [14,26,32], the amniotic membrane is not merely a structural barrier; its stromal matrix is rich in cytokines, growth factors, and anti-inflammatory proteins. These bioactive components play a crucial role in suppressing excessive fibrosis while promoting physiological tissue repair. Although Trivedi et al. suggested using the graft temporarily and removing it later [33], our long-term follow-ups and large-series data in the literature show that permanent integration of the graft is safe, it organizes under retinal layers over time, and there is no need for additional surgical trauma [16,17]. Furthermore, the fact that researchers such as Caporossi et al. and Yamamoto et al. achieved successful results with hAM even in much more severe presentations complicated by trauma or retinal detachment proves the reliability and versatility of the material [21,23]. Bokor and Fodor also stated in their review that biological scaffolds such as hAM are among the most important instruments compensating for the insufficiency of standard techniques, especially in large holes over 600 µm [1].

Our study has several limitations inherent to its design. First, it is a retrospective case series with a small sample size (n = 10) and no control group; therefore, our findings should be interpreted as preliminary. Second, although anatomical closure was achieved in all cases, the follow-up period (mean of 7 months) is relatively short to fully evaluate long-term complications. Third, there is a potential selection bias as most cases were idiopathic. Finally, functional assessment relied primarily on BCVA and structural OCT findings. Advanced functional tests such as microperimetry or multifocal electroretinography, which could have provided a more detailed evaluation of retinal sensitivity, were not available in this retrospective series and represent an important area for future prospective studies.

## 5. Conclusions

In conclusion, these preliminary results suggest that epiretinal cryopreserved human amniotic membrane transplantation is a feasible and promising salvage technique that facilitates anatomical closure, allows for functional improvement, and carries a low risk of complications in refractory macular holes in which conventional surgery has failed. By providing a biological scaffold, this technique addresses the critical challenge of the “No-ILM” environment.

However, these findings should be interpreted with caution due to the retrospective nature of the study and the small sample size. Future prospective, randomized controlled trials with larger cohorts and longer follow-up periods are necessary to validate these outcomes and to compare the efficacy of hAM transplantation directly with other techniques such as autologous retinal transplantation.

## Figures and Tables

**Table 1:** Demographics and clinical characteristics of patients.

ID	Age	Gender	Eye	Dx	Prior Surgery	AL (mm)	MH Min (µm)	MH Base (µm)	Preoperative BCVA (logMAR)	Postoperative BCVA (logMAR)
1	53	F	R	RMH	PPV + ILMp + C3F8	22.6	715	1115	1.6	1.0
2	58	M	L	RMH	PPV + ILMp + C3F8	23.7	760	1234	1.4	1.2
3	59	F	R	RMH	PPV + ILMp + C3F8	24.2	682	1087	1.2	0.8
4	50	F	L	RD + RMH	PPV + ILMp + SO	24.1	502	1205	1.8	1.4
5	63	M	R	RMH	PPV + ILMp + C3F8	23.2	730	945	1.8	1.2
6	61	M	L	RMH	PPV + ILMp + C3F8	22.3	712	1118	1.7	1.2
7	50	F	R	RMH	PPV + ILMp + C3F8	22.7	812	1302	1.6	1.4
8	68	F	R	RMH	PPV + ILMp + C3F8	23.0	612	989	1.7	1.0
9	50	F	L	RMH	PPV + ILMp + C3F8	24.0	724	1023	1.6	0.8
10	55	F	R	RMH	PPV + ILMp + C3F8	22.6	714	1145	1.6	0.8

AL: axial length; BCVA: best-corrected visual acuity; C3F8: perfluoropropane gas; Dx: diagnosis; F: female; ILMp: internal limiting membrane peeling; L: left; M: male; MH: macular hole; MH base: macular hole base diameter; MH min: macular hole minimum diameter; PPV: pars plana vitrectomy; R: right; RD: retinal detachment; RMH: refractory macular hole; SO: silicone oil.

## Data Availability

The data that support the findings of this study are available from the corresponding author upon reasonable request.

## Abbreviations

The following abbreviations are used in this manuscript:

ART	Autologous (neurosensory) Retinal Transplantation
BCVA	Best-Corrected Visual Acuity
BD	Base Diameter
bFGF	Basic Fibroblast Growth Factor
DONFL	Dissociated Optic Nerve Fiber Layer
EGF	Epidermal Growth Factor
ELM	External Limiting Membrane
EZ	Ellipsoid Zone
hAM	Human Amniotic Membrane
ILM	Internal Limiting Membrane
logMAR	Logarithm of the Minimum Angle of Resolution
MH	Macular Hole
MLD	Minimum Linear Diameter
NGF	Nerve Growth Factor
OCT	Optical Coherence Tomography
PFCL	Perfluorocarbon Liquid
PPV	Pars Plana Vitrectomy
RD	Retinal Detachment
RPE	Retinal Pigment Epithelium
SD-OCT	Spectral-Domain Optical Coherence Tomography
SO	Silicone Oil
TGF-β	Transforming Growth Factor-beta
